# Differential expression of selected histone modifier genes in human solid cancers

**DOI:** 10.1186/1471-2164-7-90

**Published:** 2006-04-25

**Authors:** Hilal Özdağ, Andrew E Teschendorff, Ahmed Ashour Ahmed, Sarah J Hyland, Cherie Blenkiron, Linda Bobrow, Abhi Veerakumarasivam, Glynn Burtt, Tanya Subkhankulova, Mark J Arends, V Peter Collins, David Bowtell, Tony Kouzarides, James D Brenton, Carlos Caldas

**Affiliations:** 1 Cancer Genomics Program, Department of Oncology, Hutchison/MRC Research Centre, University of Cambridge, Cambridge CB2 2XZ, UK; 2 Ankara University, Institute of Biotechnology, Beşevler 06500 Ankara, Turkey; 3 Molecular Histopathology, Pathology Department, Addenbrooke's Hospital, University of Cambridge Box 235, Level 3, Hills Road, Cambridge CB2 2QQ, UK; 4 Wellcome/Cancer Research UK Gurdon Institute and Department of Pathology, University of Cambridge, Tennis Court Road, Cambridge CB2 1QR, UK; 5 Cambridge NTRAC Centre, Cambridge, UK; 6 Ian Potter Centre for Cancer Genomics and Predictive Medicine, Peter MacCallum Cancer Centre, St. Andrew's Place, East Melbourne,Victoria 3002, Australia

## Abstract

**Background:**

Post-translational modification of histones resulting in chromatin remodelling plays a key role in the regulation of gene expression. Here we report characteristic patterns of expression of 12 members of 3 classes of chromatin modifier genes in 6 different cancer types: histone acetyltransferases (HATs)- *EP300, CREBBP*, and *PCAF*; histone deacetylases (HDACs)- *HDAC1, HDAC2, HDAC4, HDAC5, HDAC7A*, and *SIRT1*; and histone methyltransferases (HMTs)- *SUV39H1*and *SUV39H2*. Expression of each gene in 225 samples (135 primary tumours, 47 cancer cell lines, and 43 normal tissues) was analysedby QRT-PCR, normalized with 8 housekeeping genes, and given as a ratio by comparison with a universal reference RNA.

**Results:**

This involved a total of 13,000 PCR assays allowing for rigorous analysis by fitting a linear regression model to the data. Mutation analysis of *HDAC1, HDAC2, SUV39H1*, and *SUV39H2 *revealed only two out of 181 cancer samples (both cell lines) with significant coding-sequence alterations. Supervised analysis and Independent Component Analysis showed that expression of many of these genes was able to discriminate tumour samples from their normal counterparts. Clustering based on the normalized expression ratios of the 12 genes also showed that most samples were grouped according to tissue type. Using a linear discriminant classifier and internal cross-validation revealed that with as few as 5 of the 12 genes, *SIRT1, CREBBP, HDAC7A, HDAC5 *and *PCAF*, most samples were correctly assigned.

**Conclusion:**

The expression patterns of HATs, HDACs, and HMTs suggest these genes are important in neoplastic transformation and have characteristic patterns of expression depending on tissue of origin, with implications for potential clinical application.

## Background

Epigenetics refers to modifications in gene expression that are controlled by heritable but potentially reversible changes in DNA methylation and/or chromatin structure. Nucleosome remodelling complexes twist and slide nucleosomes in an ATP-dependent manner facilitating the accessibility of the DNA to transcription factors. Post-translational modifications of the N-terminal tails of histones within a nucleosome correlate with transcriptional regulation. Variant histones that can replace canonical histones in a nucleosome between S phases in a dynamic manner, harbour distinct information to respond to DNA damage. Methylation at the C-5 position of cytosine residues in CpG dinucleotides by DNA methyltransferases facilitates static long-term gene silencing and confers genome stability through repression of transposons and repetitive DNA elements. Perturbationof epigenetic balances may lead to alteration in gene expression, ultimately resulting in cellular transformation and tumorigenesis [reviewed in [1] and [2]].

The histone proteins that package DNA into chromatin play key roles in the regulation of transcription. The N-terminal tails of these proteins are subjected to several post-translational modifications such as acetylation, deacetylation, methylation, phosphorylation, ubiquitination, sumoylation, and ADP-ribosylation [3]. The combination of these covalent modifications gives rise to what is known as the "histone code" [4]. Transcription becomes active when histones are acetylated by histone acetyltransferases (HATs), silenced when histones are deacetylated by histone deacetylases (HDACs) and silenced or activated when methylated by histone methyltransferases (HMTs) [5]. In addition several studies have shown that chromatin modifiers regulate the expression of different sets of genes involved in tumorigenesis [6,7].

The histone acetyltransferases *EP300 *and *CREBBP *acetylateseveral lysine residues on histone proteins H2A, H2B, H3, H4, and *PCAF *acetylates histone H3. These enzymes also acetylate several non-histone proteins such as p53, β-catenin, GATA and HMGI(Y) [8,9]. Histone deacetylases are grouped into three classes based on homology to yeast histone deacetylases. Class I histone deacetylases, _*HDAC1*, *HDAC2*, *HDAC3 *and *HDAC8*_, are homologous to yeast *RPD3*. Class II histone deacetylases, _*HDAC4*, *HDAC5*, *HDAC6*, *HDAC7A*, *HDAC9*, *HDAC10*, and *HDAC11*_, share homology with yeast Hda1. The third class of human histone deacetylases has seven members, SIRT1-7, withhomology to yeast Sir2 [10].

Several lysine residues on H3 and H4 are subjected to methylation by lysinemethyltransferases and a few arginine residues are methylated by arginine methyltransferases. The histone lysine methyltransferases, *SUV39H1*and *SUV39H2 *are members of the SUV39 family of SET domain containing proteins [11]. Methylation of H3 K9 by *SUV39H1 *and *SUV39H2 *is associated with transcriptional repression. The methylation of H3 K4 by SET7/9 is associated with transcriptional activation. *EZH2*, a member of the SET1 family of HMTs, methylates H3 lysine 27, resulting in gene silencing [12]. *CARM1 *is a histonearginine methyltransferase and methylates arginine 2, 17, and 26 of H3 [13].

Several findings have suggested a role for HATs, HDACs and HMTs in cancer. *EP300 *and *CREBBP*, are fused to *MLL *in acute myeloid leukaemia [14]. *EP300 *somatic mutations coupled with the deletion of the second allele were reported in different primary tumours and cell lines [15,16]. *HDAC1 *overexpression occurs in gastric cancer [17] and modulates breast cancer progression [18]. A class 3 HDAC, *SIRT1*, was identified as an NAD-dependent p53 deacetylase [19]. InSIRT1 deficient mice, p53 hyperacetylation was observed and p53-dependent apoptosis was affected [20]. In the double knockout Suv39h1/Suv39h2 mouse the reduced level of H3 K9 methylation is associated with genomeinstability and predisposition to cancer [21]. Another indication suggesting *SUV39H1 *might be important in cancer, comes from the study revealing the interaction of SUV39H1 with Rb and also that Rb mutants found in human cancers fail to bind SUV39H1 [22]. Overexpression of *EZH2 *is associated with progression of prostate cancer and aggressiveness of breast cancer [23,24].

Epigenetic modifications appear to occur in specific patterns during neoplastic transformation. For example, a profile of CpG island hypermethylation for each tumour type allows classification using hierarchical clustering [25]. A seminal report has shown that the global loss of monoacetylation and trimethylation of histone H4 is a common hallmark of human tumor cells [26]. More recently it has also been reportedthat changes in global levels of individual histone modifications assayed at the tissue level are associated with cancer and that these changes are predictive of clinical outcome in prostate cancer [27].

Understanding the molecular details behind epigenetics and cancer holds potentially important prospects for medical treatment, and might allow the identification of new targets for drug development [1]. We carried out sequence and expression analysis of selected members of the 3 classes of histone modifier genes: HATs (*EP300, CREBBP, PCAF*), HDACs (Class I-*HDAC1, HDAC2*, Class II-*HDAC4, HDAC5, HDAC7A*, Class III-*SIRT1*), and HMTs (*SUV39H1, SUV39H2, EZH2*) in 225 samples representing 6 different solid tumour types. This represents the most comprehensive and rigorous evaluation of the profiles of chromatin modifier enzymes in human cancers done to date.

## Results

### Differential expression of histone modifier genes

The expression levels of the 12 chromatin modifier genes were analysed using QRT-PCR in 47 cancer cell lines (ovarian, breast, colorectal) and 178 primary samples: 20 colorectal tumour/normal pairs, 12 renal tumour/normal pairs, 26 breast tumours, 5 normal breast tissue samples, 45 ovarian tumours, 15 glioblastomas, 17 bladder tumours, and 6 normal bladder tissue samples. To capture intra-assay variability all QRT-PCR reactions were carried out in triplicate.

**Figure 1:**
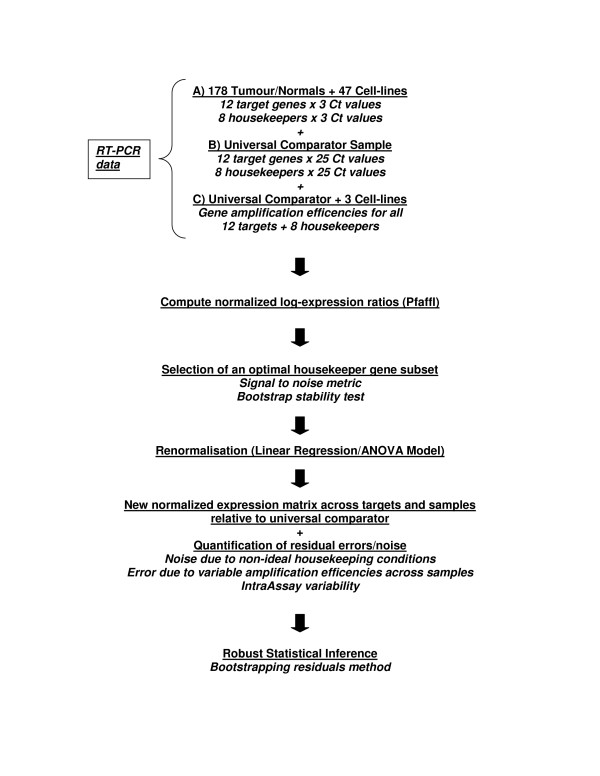
Schema of RT-PCR data analysis.

The expression data analysis strategy used is shown in schematic form in Figure 1. Normalisation of the expression levels to an endogenous housekeeping gene has been proposed [28,29] to account for sample to sample variations. The accuracy of normalising to such an internal control gene rests mainly on the assumption that this reference gene is unregulated and that it is thus constantly expressed across samples. However, as many studies have now shown, see e.g [29], traditional housekeeping genes such as *GAPDH *do show significant variability across samples. It is therefore necessary to consider a set of candidate reference genes and to choose the most stable subset for normalisation. Here we carried out RT-PCRfor a total of eight candidate housekeeping genes (*ACTB, B2M, GAPDH, HMBS, HPRT, RPL3, SDH*, and *UBC*) across the whole sample set. The normalisation was done for tissues and cell-lines separately using a three-step procedure. First, expression values were normalised to correct for variable amplification efficiencies across genes, as previouslyreported [28]. Second, we determined a subset of housekeeping genes that were stably expressed relative to the variability exhibited by the target genes. To do this we first computed gene stability measures by modifying the method of [29] to use ratios of "efficiency corrected" *Ct *values [28]. This ensured that the variability computed was less confounded by gene amplification efficiency differences across samples and not confoundedby sample loading variations [30]. We then used these measures to model thestability of candidate housekeeping genes using a randomised test of variance. We found that the expression level of housekeeping genes was morevariable in cell lines as compared to tissue samples. Thus, whereas for thetissue samples the stable subset included all eight housekeeping genes, forcell lines the stable subset did not include *B2M, HPRT*,and *RPL3*. Finally, to rigorously quantify the normalisation errors incurred we fitted a linear model to the expression ratios obtained throughstep 1 by including all stable housekeeping genes, efficiency, and replicate measurements (there are (12+8) × 178 × 3 = 10,680 measurements for tissues,and (12+5) × 47 × 3 = 2,397 measurements for cell lines). The output of the model was an estimated matrix *VG*, which contained the normalised relative expression ratios by gene (rows) and samples (columns) (see Additional file 1 and file 2). Fitting a model to data as was done here provided us with an appropriate framework in which to carry out subsequent robust inferences using a bootstrapping procedure [31,32].

**Figure 2:**
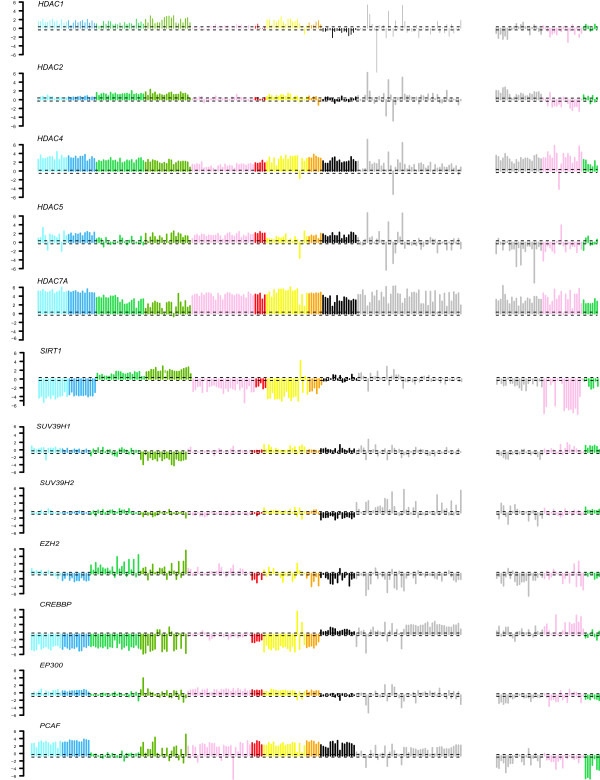
Normalized relative expression ratios of genes across all samples. Primary samples are on the left panels and grouped along the horizontal axis by tissue type according to the following colour codes: renal tumours (**LIGHT BLUE**), renal normals (**DARK BLUE**), colorectal tumours (**DARK GREEN**), colorectal normals (**LIGHT GREEN**), breast tumours (**PINK**), breast normals (**RED**), bladder tumours (**YELLOW**), bladder normals (**ORANGE**), glioblastomas (**BLACK**), ovarian tumours (**GREY**). Cell line samples are on the right panels and are also grouped by tissue type: ovarian (**GREY**), breast (**PINK**) and colorectal (**DARK GREEN**). The y axis shows the expression ratios on a log2 scale. The horizontal dashed lines represent an averaged one standard deviation (-0.4 to 0.4) gaussian noise level arising from unstable housekeeping gene expression across the whole sample set. The vertical distance between the two dashed lines represents therefore a zero centred 70% average confidence interval for all the expression values.

Figure 2 shows the normalized relative expression ratios derived from the model for each of the 12 histone modifier genes analysed (see also Table 1). Inspection of this figure provides an overview of expression of each of the genes analysed across all samples. For example, *HDAC1 *overexpression was seen in renal, bladder, colorectal tumour and normal tissues, and a small proportion of ovarian primary tumours. In contrast underexpression was seen in most of the glioblastomas, 25% of the primary ovarian tumours and about 1/3 of the ovarian cell lines, and most breast cancer cell lines. In normal breast tissues and primary breast cancers *HDAC1 *expression changes were mostly not significant.

One important aspect was to determine which genes could be used to differentiate between tumour and normal tissues based on expression analysis (Table 2). Inspection of the data from the paired and unpaired tissues suggested for example differential expression of *HDAC5, SIRT1, SUV39H1 *and *EZH2 *(Figure 2). To test this, we used the non-parametric Wilcoxon rank sum test as it makes no assumptions about the distribution of expression values within tissue types and is robust to possible unrepresentative outliers in the tissue sets. To further check therobustness of the p-values from the rank sum test we used the bootstrappingresidual method [31,32] to model noise due to unstable housekeeping gene expression. We therefore generated an additional 99 *VG *matrices representing perturbations around the estimated *VG*. A robustness measure for each tumour/normal tissue pair p-value was then obtained as thenumber of times (out of 100) the test was significant at a 0.001 significance level (Table 2). This showed that colorectal tumours were distinguished as a group from normal colorectal tissues by the expression of *HDAC1, HDAC5, HDAC7A*, *SIRT1*, and *SUV39H1*. In pairwise comparisons, all colorectal cancers showed significantly lower expression (P < 0.001) of *HDAC1*, *HDAC5*, and *SIRT1*,than their respective normals, except for two colorectal tumours showing higher expression of *HDAC5*. Higher expression of *HDAC7A *and *SUV39H1 *was observed in most colorectal tumours. However, 3 colorectal tumours showed lower expression of *HDAC7A*. Renal tumours were distinguished as a group from normal renal tissues by the expressionof *EZH2 *(*PCAF *was useful in distinguishing the two groupsin less than 50% of the simulations). In pairwise comparisons with their matched normal tissue all renal tumours expressed higher levels of *EZH2*. Breast tumours were distinguished as a group from normal breast tissues by the expression of *EZH2*, *CREBBP *and *HDAC4*. Although the number of normal breast samples available was small the analysis is robust statistically and it is reassuring to see thatfor the single gene (EZH2) out of the 3 that are discriminatory and for which independent data exists the results are concordant with our findings (EZH2 is overexpressed in cancers vs normals) [24]. Bladder tumours could not be distinguished as a group from the bladder normal tissues based on the individual expression of any of the genes analysed. Further insight however was obtained by application of Independent Component Analysis [33] (see later).

### Histone modifier genes have tissue-type specific patterns of expression

**Figure 3:**
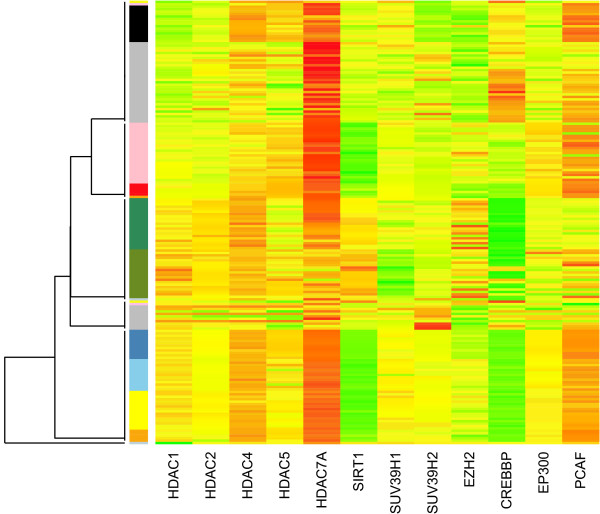
Cluster analysis of expression matrix of 12 genes across primary samples using the ensemble learning algorithm. Red denotes overexpression, green underexpression. See Figure 2 for detailed expression values.

We also noted what appeared to be distinct expression profiles for each tissue type (for example compare expression of *CREBBP *in glioblastomas versus renal cancers). To investigate this further we clustered the samples based on the similarity of expression across genes and then visualized the data in a matrix format. We first used unsupervised approaches because we were interested in discovering novel associations without influence from prior knowledge. Unsupervised algorithms that have been used extensively for expression analysis include hierarchical clustering [34] and k-means [35]. However, both have limitations: k-means is biased as it requires the number of clusters to be specified in advance whereas hierarchical clustering does not allow this number to be rigorouslyinferred. The problem of inferring the number of clusters has been addressed [36] in the context of a Gaussian mixture model. There the Bayesian Information Criterion (BIC) was used to infer the number of clusters. An alternative to BIC is provided by the variational Bayesian approach [37]. This approach implements an ensemble learning algorithm for the cluster parameters and provides a rigorous framework in which to infer the optimal number of clusters [38] (see *Methods*). Moreover, in common with the method in [39] it provides a framework in which to test the robustness ofthe clusters to noise. Prior knowledge may be easily incorporated, althoughfor this unsupervised analysis we have implemented a version with complete uninformative priors. The results of unsupervised clustering using the ensemble-learning algorithm as applied to the normalised expression matrix *VG *are summarised in Table 3. On the set of 12 target genes, the algorithm predicted the presence of six clusters (Figure 3). One included most breast samples, a second included the renal and bladder samples, and athird included most colorectal samples. The ovarian samples were distributed between two main clusters (a third cluster contained a single case), one of which shared with glioblastomas. We compared this clustering pattern with the one obtained using hierarchical clustering and found that the patterns were mostly concordant (see Additional file 3).

New insights were obtained using Independent Component Analysis [33], whichis described in detail in Methods. The aim here was two-fold. One goal was to find in an unsupervised manner data projections that may be of specific biological interest and to find the major players (genes) defining these projections. Secondly, ICA allows the inherent dimensionality of the data set to be inferred via a dimensional reduction step in which Gaussian noise-like dimensions are filtered out [33]. Applying a maximum likelihood version of ICA we were able to infer only seven robust projections or modes. Thus, ICA removed a five dimensional gene subspace for which the data variance was smallest and along which the data distribution was Gaussian. Out of the seven modes, four were particularly interesting (see Additional file 4-a) clearly discriminating the various tumour types from each other or from their normal counterparts. For example, ML-IC7 showed a projection that separated tumour from normal tissues across four different tissue types(Breast, Renal, Bladder and Colorectal), which we verified with a Wilcoxon rank sum test (p-values were 2 × 10e-5, 3 × 10e-5, 2 × 10e-3 and 1 × 10e-2,respectively). Taken together with its corresponding projection along genes(see Additional file 4-b) this mode defines a pattern of relative over-and-underactivation of the twelve genes that discriminates tumours from normals and that may have biological significance. Similarly, the other modes (see Additional file 4) suggested that *SIRT1 *and *CREBBP *to be among the top genes discriminating the various tissue types. An ensemble learning clustering over the four genes with the best signal tonoise ratios (see Additional file 4-c) confirmed that even with a small number of genes we could separate tissue types from each other.

The unsupervised clustering results strongly suggest that cancer tissues may be distinguished from each other on the basis of the expression profiles of 12 or less chromatin modifier genes. Many classification algorithms exist and have been applied extensively to gene expression data (see [40,41] and [42] for an overview). Because of the relatively large number of classes (6 tissue types) and the small number of predictors (12 target genes) our classification problem is well suited for a parametric mixture model based approach [42,43]. Here we adapted the variational Bayesian Gaussian mixturemodel to the supervised setting. To ensure robustness of the results to noise we restricted the classifier to be in a seven dimensional gene subspace spanned by the genes with the best overall signal to noise ratios (*HDAC5, HDAC7A, SIRT1, SUV39H1, EZH2, CREBBP, PCAF*). Two methods of internal cross-validation were used to partition the sample set into training and test sets. In the leave-one-out method, one sample from each tissue type was selected at random and placed in the test set. In the second method we placed 20% of randomly selected samples from each type in the test set. For a given classifier we learned from the training set the meansand variances of the clusters associated with each tissue type. This was done on a tissue-type basis. We then assigned the test samples to a tissue type using a linear discriminant classifier (see Methods). The error rates of the classifier on the training and test sets were recorded. This was then repeated for 1000 different randomly selected partitions of the sample set into training and test subsets. The average and standard deviation of theerror rates over these 1000 runs were then computed. Finally, all these steps were repeated for all possible numbers and combinations of genes out of the initial set of seven. That is, for each possible subset of (*HDAC5, HDAC7A, SIRT1, SUV39H1, EZH2, CREBBP, PCAF*) containing at least two genes (a total of 1 + 7 + 2 × 21 + 2 × 35 = 120 subsets) we did the analysis described above recording the average error rate on the test set together with its standard deviation (Table 4). From the classificationresults (Table 4) we found that based on this data set we can very accurately predict tissue type on the basis of very few genes. With as few as three genes (*SIRT1, CREBBP, HDAC7A*) we can obtain prediction rates over 80%. Moreover, we can see (Table 4) that in fact many optimal classifiers exist. One possible choice would be the classifier (*SIRT1, CREBBP, HDAC7A, HDAC5, PCAF*), which gave averageprediction rates of 87% and 86% for the training and test sets, respectively. Using all 12 target genes in the classifier we obtained 92% ± 1% and 86% ± 5% prediction rates for the training and test sets, respectively. We found however this last result not to be robust to noise arising from non-ideal housekeeping gene conditions which is why we focused on the genes with best signal-to-noise ratios. To test our classifier(s) further we validatedour results against 86 independent breast tumour samples, which became available after our initial analysis. We found that with the optimal two-gene classifier (*SIRT1, CREBBP*) about 80% of these independent breast tumour samples could be correctly classified. This classifier's prediction rate on the training set was 76% (training set) and 74% (internaltest set) respectively.

Even though the accuracy and reproducibility of microarray experiments is questionable, particularly, when the focus is on a small number of genes, we decided to test our results further by studying the expression profiles of our chromatin modifier genes in an external independent microarray data set [44]. Out of the 12 histone modifier genes studied using RT-PCR there were 10 that were profiled in this microarray study (*SUV39H2 *and *HDAC7A *were not present on the array platform used) across many different cancer types including 34 breast, 13 renal, 23 colorectal and 50 ovary samples. We first applied the Wilcoxon rank sum test to see whether the 10 genes profiled in [44] could discriminate any of these four tissue types from each other (6 pairwise comparisons). We found that many of the genes were discriminatory, yet when compared with our study the number of genes discriminating any given pair of tissue types was significantly smaller (see Additional file 5). Thus, for a given pair of tissue types thenumber of discriminatory genes varied from 2 to 4 (out of a possible 10), whilst for our study this number varied from 7 to 11 (out of a possible 12). Applying, on the microarray data, the same classification algorithm and internal cross validation as before, showed that the genes were not able toconsistently classify samples according to tissue type (error rates were over 50% when classifying with all 10 genes, the six discriminatory genes (see Additional file 5 ), or with our optimal 4-gene classifier (*SIRT1,CREBBP,HDAC5 *and *PCAF*)). However, when we considered classifying only two tissue types at a time, we obtained much better classification rates. Thus, using internal cross validation with a 20% test set partition and using the discriminatory genes as classifier genes we found in some cases excellent prediction rates. For example, usingthe classifier (*HDAC1, HDAC2, HDAC4, EZH2*) we obtained 94% prediction rates for discriminating colorectal from renal tumour samples. We confirmed this by unsupervised clustering which clearly separated colorectal from renal tumours (data not shown). In summary, theseanalyses support the existence of tissue-specific patterns of expression ofchromatin modifier genes.

### Mutations of HDAC1, SUV39H1, and SUV39H2 in epithelial cancers are rare

We also screened *HDAC1, HDAC2, SUV39H1*, and *SUV39H2 *for mutations in 65 cancer cell lines and 116 primary tumours. Themutations and sequence alterations identified in these genes are summarizedin Tables 5 and 6.

*HDAC1 *was analysed by SSCP, and a silent polymorphism was identified in one breast tumour sample.

*HDAC2 *was analysed with both SSCP and DHPLC. A single nucleotide deletion was found in a colorectal cancer cell line (HCT15), causing a frameshift starting at amino acid 543 of the protein and resulting in the addition of 16 amino acids to its C-terminal. A insertion of a CAG triplet was identified in the 5'UTR at nucleotide 143 (position -37 from ATG) in 18% of the cancer samples. This insertion was shown to be germline in all samples for which matched normal DNA was available for testing. This 5'UTR alteration was found using capillary electrophoresis in only 10% of 192 normal DNA controls (p < 0.01, Fisher's exact test). No correlation was foundbetween the CAG insertion and expression levels of HDAC2 (data not shown). In addition four cancer samples with intronic polymorphisms were also identified.

*SUV39H1 *was analysed by SSCP and Capillary Electrophoresis based Heteroduplex Analysis (CEHA). A nonsense mutation 862C>T causing the disruption of the protein's SET domain (Q288STOP), was found in one ovariancancer cell line (UCI101). A silent polymorphism and an intronic sequence variant were also identified.

*SUV39H2 *was screened by SSCP. An insertion of a single T in the 5'UTR (nucleotide 52 of cDNA Accession number NM_024670, nucleotide -14 from start codon) was found in a primary breast tumour. This alteration wassomatic. A missense sequence alteration, R74Q (442A>C), was identified in 4% of the cancer samples. This alteration was proven to be germline in the 5 primary tumours where normal tissue was available for testing, and represents a probable polymorphism. Two silent polymorphisms were also identified.

## Discussion

The rationale to study the alterations of chromatin modifier genes in cancer samples and their respective normal tissues seemed obvious to us given the biology and the previous indications for their involvement in tumorigenesis. The mutational analysis reported here, and previous work by our group and others, shows that inactivating mutations of histone modifiers are rare, although *EP300 *and *CREBBP *are targets of chromosomal translocations in human leukaemias and *EP300 *and *CREBBP *are an uncommon target of mutations in epithelial cancers [13-15,45-47]. A finding that needs confirmation in a larger association study isthe observation that the CAG insertion identified in the 5'UTR of *HDAC2 *could be associated with cancer predisposition.

The expression profile of selected chromatin remodelling genes from the three classes of histone modifiers was analysed in a large sample panel. This represents the most comprehensive analysis of the expression alterations of these important genes in human cancers and their corresponding normal tissues. The analysis was done rigorously with normalization of expression levels in comparison with several stable housekeeping genes and in relation to a universal reference RNA. By fittinga linear regression model to the data we could quantify the residual error due to unstable housekeeping gene expression and determine that the expression levels of the 12 chromatin modifier genes varied significantly across samples. The main findings of the analysis were: 1- that there are tissue-specific histone-modifier gene expression signatures (some constituted by as few as 3 to 5 genes); 2- that for certain tissue types there are significant expression changes between normal and malignant cells;and 3- that expression patterns in cell lines are frequently significantlydifferent from the corresponding primary tumours.

The existence of characteristic histone modifier gene expression signaturesin different tissues is a remarkable finding particularly when taken in thecontext of the recent reports of global and characteristic changes in histone modification in cancer [26,27]. Ensemble learning and hierarchical clustering algorithms applied on the normalized expression ratios of the 12 chromatin remodelling genes successfully separated the tumour samples according to their tissue types (Figure 3). We verified that the clusters obtained using the ensemble learning algorithm are robust to both the algorithm initialisation point and the error due to unstable housekeeping gene expression. This was done rigorously by bootstrapping residuals in thelinear model [37,33] and building consensus groups over a large number (~1000) of clustering runs. As few as five genes (*SIRT1, CREBBP, PCAF, HDAC7A, HDAC5*) were informative enough to group the samples successfully according to tissue type (Table 4). In an independent microarray data set we found that these chromatin modifier genes were also able to discriminate samples according to tissue type, although the degree of discrimination was much smaller. These findings suggest a mechanistic link between the gene expression changes reported here and global tumour-specific histone modifications reported by others.

The expression levels of some of the genes could also be used to distinguish between tumour and the respective normal tissue. *HDAC1, HDAC5, HDAC7A, SIRT1*, and *SUV39H1 *expression profiles were distinctive for colorectal cancers and normal colorectal mucosa. *EZH2 *expression was found to be informative in distinguishing renal tumour and normal renal tissue pairs, and also breast tumours from breast normal tissues. Breast tumours and normals were also distinguished by the expression profile of *HDAC4 *and *CREBBP*. Using ICA we alsofound a pattern of relative expression over all 12 genes (ML-IC7, see Additional file 4) that is able to discriminate tumours from normals acrossfour different tissue types (Breast, Colorectal, Renal and Bladder). These findings raise the prospect that there will be a therapeutic index when using drugs that target these enzymes in the clinic.

Comparison of normalized expression ratios of tumours with their relevant cancer cell lines revealed significant differences highlighting some of theproblems of using cell lines as models of cancer (Figure 2). Breast cell lines showed downregulation of *HDAC1, HDAC2, HDAC5, EZH2, EP300*, and *PCAF *compared to breast tumours. *CREBBP *and *SUV39H1 *upregulation was observed in breast cell lines compared tobreast tumours. Colorectal cell lines showed *SIRT1, EZH2, PCAF *underexpression and *SUV39H1 *overexpression compared to colorectal tumours. *PCAF *downregulation was seen in ovarian cell lines compared to ovarian tumours. This raises problematic questions about using cell lines to model primary tumours, for example when doing HDAC inhibitor compound screening.

Chromatin remodelling genes and their involvement in transcriptional regulation has been the focus of previous studies although none as systematic as what we report here. Overexpression of *HDAC1 *has been seen in gastric and breast cancers [17,18]. In our study we did not observe significant expression changes of *HDAC1 *when comparing tumour and normal tissue samples, except for colorectal cancers. *EZH2 *overexpression was previously seen in prostate cancer [23]. Subsequently, it was shown that EZH2 overexpression was associated with the aggressivenessof breast cancer [24]. Our results confirm that overexpression of *EZH2 *is found in breast tumours compared to the normal breast samples and shows for the first time *EZH2 *overexpression in renal tumours. Overexpression of HDAC2 was recently reported in colon cancer [48]. In our series *HDAC2 *overexpression was observed in 50% of colorectal tumours compared to their normal pairs.

## Conclusion

Our findings have implications for tumour biology, differences in histone modifications between tumour types and the application of histone-modification-altering drugs. Ongoing work aims at correlating histone modifier gene expression with global histone modification patterns and obtaining a more systematic analysis of all known histone modifiers enzymes using custom gene arrays.

## Methods

### Primary tumours and normal samples

Mutation analysis was performed on 59 primary breast tumours, 37 primary ovarian tumours, and 20 colorectal tumours. QRT-PCR analysis was done on RNA samples from 20 colorectal tumour/normal pairs, 12 renal tumour/normal pair, 27 breast tumours, 5 normal breast tissues, 17 bladder tumours, 5 normal bladder tissue, 45 ovarian tumours, and 15 glioblastomas. A second validation series of 86 primary breast cancers was subsequently profiled. Primary tumours were collected at Derby City General Hospital, Addenbrooke's Hospital, Essex County Hospital, and Freeman Hospital, Newcastle Upon Tyne. In all cases the collection of material was done with Local Research Ethics Committee approval. All tumours were 'flash' frozen immediately following surgery.

### Cell lines

Mutation analysis was performed on 65 cancer cell lines (30 ovarian, 18 breast, 4 lung, 8 pancreatic and 5 colorectal). QRT-PCR was performed on 47cancer cell lines (21 ovarian, 19 breast, 7 colorectal). Cell lines were obtained from ATCC and ECACC or as a gift from collaborating laboratories (see Additional file 6).

### Normal control samples

Normal control DNA samples (isolated from lymphoblastoid cell lines generated from apparently healthy randomly selected individuals) were obtained from ECACC (Human Random Control DNA Panel, HRC-1 and HRC-2).

### DNA isolation

Frozen primary tumours were serially sectioned onto slides. Tumour tissue was microdissected away from normal tissue and DNA extracted by SDS-proteinase K digestion. Germ-line DNA was prepared from either a matching blood sample or from normal tissue microdissected away from tumourtissue. Cell line DNA was extracted by either proteinase K or DNAzol™ (Gibco BRL).

### DNA PCR

*HDAC1 *was amplified in 15 fragments, *HDAC2 *was amplified in 13 fragments, *SUV39H1 *was amplified in 8 fragments, and *SUV39H2 *was amplified in 7 fragments of approximately 200–400 bp covering the exons and exon-intron boundaries (Primer sequences is providedin Additional file 7). Amplification reactions (30 μl) contained 20 mM (NH_4_)2SO_4_, 75 mM TrisHCl, pH 9.0 at 25°C, 0.1% (w/v) Tween,2.5–3 mM MgCl_2_, 200 mM dNTP, 10pmoles of each primer and 2.5 U of Red Hot DNA polymerase (Advanced Biotechnologies). The amplifications were doneusing a DNA Engine Tetrad, MJ Research PTC-225 Peltier Thermal Cycler.

### Single Strand Conformation Polymorphism/Heteroduplex Analysis (SSCP/HA)

*HDAC1 *and *SUV39H2 *were analysed by SSCP/HA. Formamide loading buffer was added to PCR products. The mix was denatured at 95°C for 10 minutes and kept on ice until loading onto 0.8XMDE (Mutation Detection Enhancement) gel (Flowgen). Gels were run overnight at 120V and 4°C.

### Denaturing High Performance Liquid Chromatography (DHPLC)

*HDAC2 *was analysed by DHPLC. PCR products were denatured at 95°C for 5 minutes and cooled down -1°C/cycle to 30°C. PCR products of 8 samples were pooled and injected in the Transgenomics WAVE DHPLC using 3 different temperatures. Melting temperatures were calculated with the DNA Melt program [49].

### Capillary Electrophoresis based Heteroduplex Analysis (CEHA)

*SUV39H1 *was analysed by CEHA. PCRs were carried out using 10 pmol of 5'FAM labelled M13 forward primer 3 pmol of sequence specific forward primer with an M13 sequence tail and 10 pmol of sequence specific reverse primer. PCR products of samples were mixed with control PCR products denatured 10 min. at 95°C and cooled down -1°C/cycle to 30°C.PCR products were diluted 1/10 in water mixed with 0.3 μl of GS500 size standard and run on ABI3100 on GeneScan Polymer (5%GSP (ABI), 10% Glycerol and 1XTBE) at 25°C.

### Capillary Electrophoresis

The presence of *HDAC2 *CAG repeat insertion was investigated in control DNA samples by capillary electrophoresis. A new primer pair was designed for an amplicon of 112 bp comprising the CAG repeat. PCR products were run on ABI3100 genetic analyser on ABI POP-6 polymer (Applied Biosystems, Foster, CA, USA). Size analysis was done on GeneScan Analysis 3.7 software.

### DNA sequencing

Purified PCR products were sequenced using ABI Prism^R ^*BigDye *terminators and an ABI3100 genetic analyser (Applied Biosystems, Foster, CA, USA). All samples with a mutation were re-amplified and re-sequenced.

### RNA isolation

Total RNA was isolated from primary tumours and cancer cell lines using Trizol reagent (Gibco BRL).

### cDNA synthesis and real time PCR

cDNAs were synthesized by reverse transcription of 2 μg total RNA usingrandom hexamers. Real Time PCR was carried out using SYBR Green PCR Master Mix (Applied Biosystems) on an ABI 7900 Sequence Detection System (Applied Biosystems). The specificity of the PCR products was confirmed by melting curve analysis. The primer sequences for the 12 chromatin modifier genes (*HDAC1*, *HDAC2*, *HDAC4*, *HDAC5*, *HDAC7A*, *Sirt1*, *SUV39H1*, *SUV39H2*, *EZH2*, *EP300*, *CBP*, *PCAF*) and the 8 housekeeping genes (*ACTB, B2M, GAPDH, HMBS, HPRT, RPL3, SDH, UBC*) is provided in Additional file 8. Standard curves were used to determine the amplification efficiencies of the 20 genes across 4 test samples as described previously [28]. The normalized expression values of genes in individual samples were determined relative to a common comparator RNA (using formula described in 28) isolated from an immortalized B-lymphocyte cell line. The lymphoblastoid cell line was selected to generate a universal comparator RNA because it represents an inexhaustible source of RNA, and also because we verified that expression of both housekeeping genes and target genes were very stable and reproducible, with low intra and inter assay variability (in a set of 25 independent amplifications for all 12 target genes and 8 housekeeping genes).

### Expression ratios

Following Pfaffl [28] the ratio of expression of target gene *t *in sample *s *relative to our control sample *c *is given by Rtsr=⋅EtCttc−CttsErCtrc−Ctrs
 MathType@MTEF@5@5@+=feaafiart1ev1aaatCvAUfKttLearuWrP9MDH5MBPbIqV92AaeXatLxBI9gBaebbnrfifHhDYfgasaacH8akY=wiFfYdH8Gipec8Eeeu0xXdbba9frFj0=OqFfea0dXdd9vqai=hGuQ8kuc9pgc9s8qqaq=dirpe0xb9q8qiLsFr0=vr0=vr0dc8meaabaqaciaacaGaaeqabaqabeGadaaakeaacqWGsbGudaWgaaWcbaGaemiDaqNaem4CamNaemOCaihabeaakiabg2da9iabgwSixxaabeqaceaaaeaacqWGfbqrdaqhaaWcbaGaemiDaqhabaGaem4qamKaemiDaq3aaSbaaWqaaiabdsha0jabdogaJbqabaWccqGHsislcqWGdbWqcqWG0baDdaWgaaadbaGaemiDaqNaem4CamhabeaaaaaakeaacqWGfbqrdaqhaaWcbaGaemOCaihabaGaem4qamKaemiDaq3aaSbaaWqaaiabdkhaYjabdogaJbqabaWccqGHsislcqWGdbWqcqWG0baDdaWgaaadbaGaemOCaiNaem4Camhabeaaaaaaaaaa@52FD@, where *r *labels the reference gene used for normalisation. This formula corrects for variable amplification efficiencies across genes as well as correcting for unwanted sample-to-sample variation (such as RNA quality), but is only an approximation and makes two important assumptions:(i) that the reference gene has the same expression in both samples and (ii) that the amplification efficiency is also the same between the two samples. To gauge the error incurred by assumption (ii) we measured the amplification efficiency of all genes in three cell-lines in addition to our universal comparator, thus yielding four efficiency measurements labelled in what follows as *e *(see Additional file 9).

### Housekeeping gene selection

To evaluate whether a candidate housekeeping gene is suitable for normalisation we must compare its variability in expression with that of the target genes. For this purpose we defined, for each reference target genepair (*r,t*), an F-statistic [50] that can be interpreted as a signal-to-noise ratio StNr
 MathType@MTEF@5@5@+=feaafiart1ev1aaatCvAUfKttLearuWrP9MDH5MBPbIqV92AaeXatLxBI9gBaebbnrfifHhDYfgasaacH8akY=wiFfYdH8Gipec8Eeeu0xXdbba9frFj0=OqFfea0dXdd9vqai=hGuQ8kuc9pgc9s8qqaq=dirpe0xb9q8qiLsFr0=vr0=vr0dc8meaabaqaciaacaGaaeqabaqabeGadaaakeaadaWcaaqaaiabdofatnaaBaaaleaacqWG0baDaeqaaaGcbaGaemOta40aaSbaaSqaaiabdkhaYbqabaaaaaaa@3250@. The statistic evaluates whether the housekeeping gene *r *is stably expressed relative to the variability of the target gene *t*, and is defined by

Ftr=StNr=nr−1nr∑r′Vtr′∑r′≠rVrr′
 MathType@MTEF@5@5@+=feaafiart1ev1aaatCvAUfKttLearuWrP9MDH5MBPbIqV92AaeXatLxBI9gBaebbnrfifHhDYfgasaacH8akY=wiFfYdH8Gipec8Eeeu0xXdbba9frFj0=OqFfea0dXdd9vqai=hGuQ8kuc9pgc9s8qqaq=dirpe0xb9q8qiLsFr0=vr0=vr0dc8meaabaqaciaacaGaaeqabaqabeGadaaakeaacqWGgbGrdaWgaaWcbaGaemiDaqNaemOCaihabeaakiabg2da9maalaaabaGaem4uam1aaSbaaSqaaiabdsha0bqabaaakeaacqWGobGtdaWgaaWcbaGaemOCaihabeaaaaGccqGH9aqpdaWcaaqaaiabd6gaUnaaBaaaleaacqWGYbGCaeqaaOGaeyOeI0IaeGymaedabaGaemOBa42aaSbaaSqaaiabdkhaYbqabaaaaOWaaSaaaeaadaaeqbqaaiabdAfawnaaBaaaleaacqWG0baDcuWGYbGCgaqbaaqabaaabaGafmOCaiNbauaaaeqaniabggHiLdaakeaadaaeqbqaaiabdAfawnaaBaaaleaacqWGYbGCcuWGYbGCgaqbaaqabaaabaGafmOCaiNbauaacqGHGjsUcqWGYbGCaeqaniabggHiLdaaaaaa@538E@

where *n*_*r *_is the number of candidate housekeeping genes, *V_tr_*, denotes the sample variance of the log-ratios across samples for target gene *t *as measured by reference gene *r*' and *V_rr' _*denotes the sample variance ofthe log-ratios across samples for reference gene *r *as measured by reference gene *r*'. To motivate the above formula it is important to realise that the variability of any gene (be it a target or reference gene)can only be evaluated by comparison with another "housekeeping" gene. Thus, if two reference genes are true housekeepers than their *V_rr' _*term will be small. Thus, if the above statistic is larger than one then the target gene shows more variability than the reference gene. Confidence intervals for the statistic were found by performing a large number of bootstraps, where in each bootstrap reference genes were sampled with replacement in the denominator and numerator separately [50], and recomputing the statistic for each bootstrap. Over 5000 bootstraps were performed to obtain 95% confidence intervals (CI) for each target and reference gene pair. For a given target gene, those reference genes for which their 95% CI did not include the threshold value 1 were declared as stable relative to that target gene. Reference genes were then ranked according to the number of target genes relative to which they were stably expressed. Finally, the number of reference genes used for downstream analysis was determined by requiring a certain minimum number of target genes relative to which the reference genes were all stably expressed. To ensure reliable inferences for all target genes we developed a linear model based normalisation (see Additional file 9).

### Normalization

Out of the eight candidate housekeeping genes we selected a subset that were stably expressed relative to the variability exhibited by the target genes. The subset was chosen using the randomised variance test explained above. We then normalised the PCR data relative to this stable subset of housekeeping genes by fitting a linear regression model to the log base two ratio values

log_2_*R*_*tsre*,*i*_= *μ *+ *G*_*t*_+*V*_*s*_+ *R*_*r *_+*E*_*e *_+ (*VG'*)_*st *_+ (*VR*)_*sr *_+ (*GR*)_*tr *_+ (*EG*)_*et *_+ (*ER*)_*er *_+ (*VE*)_*se *_+ *ε*_*tsre*,*i*_

where  is the expression ratio [28] and where *t*, *s*, *r*, *i *label the target gene, sample, reference gene and replicate (i = 1,...,9), respectively. (We combined the triplicate *Ct *values to generate a set of nine replicates using a bootstrapping approach.). In the above, *E*_*te *_and *E*_*re *_denote the efficiencies of target gene *t *and reference gene *r *for sample *e*, as explained previously. The terms in the linear model represent the singleton and interaction effects as commonly defined in linear regression analysis. Thus, *μ *is the overall mean of the log-ratios, *G*_*t *_is the expression of target gene *t *averaged over all other factors and *VG'_st _*is the specific sample-gene interaction. All other terms are defined similarly. The only random term inthis model is ε and represents a Gaussian noise term. The parameters were estimated using maximum likelihood subject to the constraints

∑tGt=∑sVs=∑eEe=∑rRr=0,∑sVG′st=∑tVG′st=0,
 MathType@MTEF@5@5@+=feaafiart1ev1aaatCvAUfKttLearuWrP9MDH5MBPbIqV92AaeXatLxBI9gBaebbnrfifHhDYfgasaacH8akY=wiFfYdH8Gipec8Eeeu0xXdbba9frFj0=OqFfea0dXdd9vqai=hGuQ8kuc9pgc9s8qqaq=dirpe0xb9q8qiLsFr0=vr0=vr0dc8meaabaqaciaacaGaaeqabaqabeGadaaakeaadaaeqbqaaiabdEeahnaaBaaaleaacqWG0baDaeqaaaqaaiabdsha0bqab0GaeyyeIuoakiabg2da9maaqafabaGaemOvay1aaSbaaSqaaiabdohaZbqabaaabaGaem4CamhabeqdcqGHris5aOGaeyypa0ZaaabuaeaacqWGfbqrdaWgaaWcbaGaemyzaugabeaaaeaacqWGLbqzaeqaniabggHiLdGccqGH9aqpdaaeqbqaaiabdkfasnaaBaaaleaacqWGYbGCaeqaaaqaaiabdkhaYbqab0GaeyyeIuoakiabg2da9iabicdaWiabcYcaSmaaqafabaGaemOvayLafm4raCKbauaadaWgaaWcbaGaem4CamNaemiDaqhabeaaaeaacqWGZbWCaeqaniabggHiLdGccqGH9aqpdaaeqbqaaiabdAfawjqbdEeahzaafaWaaSbaaSqaaiabdohaZjabdsha0bqabaaabaGaemiDaqhabeqdcqGHris5aOGaeyypa0JaeGimaaJaeiilaWcaaa@615C@

and similarly for all the other interaction terms. The estimation was carried out in a robust fashion by assigning zero weights to outliers. The new normalised expression values of target genes across samples relative tothe control are given by the matrix *VG*_*st *_= *μ *+ *V*_*s *_+ *G*_*t*+ _*VG'*_*st*_. This linear model approach allows rigorous quantification of the error incurred in the normalisation due to unstable housekeeping gene expression and variable sample efficiencies through the simultaneous estimation of *VR *and *VE*.

To test the robustness of our inferences to noise arising from non-ideal housekeeping gene conditions we fitted the alternative model with *VR *= 0. We then applied the bootstrapping residual method of [31,32]to obtain a new estimated matrix *VG*, that represents a perturbation around the original *VG*. A standard error estimate for the noisearising due to non-constant housekeeping gene expression was obtained by the sample variance of the residuals in the model with *VR *= 0 (see Additional file 9). Software written in the R-language [51] that implements the normalisation as described here is available on request.

### Clustering

Clustering was done in an unsupervised fashion using an ensemble learning gaussian mixture model [reviewed in [37]]. This is a variational bayesian procedure that allows one to objectively compare mixture models with different number of clusters. This is a main advantage over other unsupervised clustering procedures such as hierarchical clustering, k-meansor SOM where the number of clusters that best describe the data cannot be reliably inferred. Inference is carried out using an optimal separable approximation to the true posterior density as explained in [37].

For our model with parameters Θ the true posterior is the product of the likelihood function

p(D|Θ)=∏n=1Np(xn|Θ)=∏n=1N∑c=1KπcG(xn|μc,Ωc)
 MathType@MTEF@5@5@+=feaafiart1ev1aaatCvAUfKttLearuWrP9MDH5MBPbIqV92AaeXatLxBI9gBaebbnrfifHhDYfgasaacH8akY=wiFfYdH8Gipec8Eeeu0xXdbba9frFj0=OqFfea0dXdd9vqai=hGuQ8kuc9pgc9s8qqaq=dirpe0xb9q8qiLsFr0=vr0=vr0dc8meaabaqaciaacaGaaeqabaqabeGadaaakeaacqWGWbaCdaqadaqaaiabdseaejabcYha8HGaciab=H5arbGaayjkaiaawMcaaiabg2da9maarahabaGaemiCaa3aaeWaaeaacqWG4baEdaWgaaWcbaGaemOBa4gabeaakiabcYha8jab=H5arbGaayjkaiaawMcaaaWcbaGaemOBa4Maeyypa0JaeGymaedabaGaemOta4eaniabg+GivdGccqGH9aqpdaqeWbqaamaaqahabaGae8hWda3aaSbaaSqaaiabdogaJbqabaGccqWGhbWrdaqadaqaaiabdIha4naaBaaaleaacqWGUbGBaeqaaOGaeiiFaWNae8hVd02aaSbaaSqaaiabdogaJbqabaGccqGGSaalcqWFPoWvdaWgaaWcbaGaem4yamgabeaaaOGaayjkaiaawMcaaaWcbaGaem4yamMaeyypa0JaeGymaedabaGaem4saSeaniabggHiLdaaleaacqWGUbGBcqGH9aqpcqaIXaqmaeaacqWGobGta0Gaey4dIunaaaa@6455@

and the priors for *μ*_*c*_, Ω_*c*_, *π*_*c*_. We used a Gaussian, Gamma and Dirichlet prior distributions for these, respectively. In the above, *N *denotes thenumber of samples to be clustered, *K *the maximum number of components to try to infer, *c *labels the component, *D *= {*x*_*n*_:*n *= 1....*N*} is the data where each *x*_*n*_∈ *R*^*d *^(*d *equals the dimension of the gene space over which clustering is done), {*μ*_*c*_, Ω_*c*_, *π*_*c*_} are the parameters to be inferred, {*μ*_*c*_, Ω_*c*_} denote the mean vector and inverse covariance matrix of the Gaussian component *c*, and *π*_*c *_denotes the weight of component *c*. One hundred optimisation runs were performed with different ensemble initialisations and the one maximising the evidence bound [37] was selected. The number of clusters andcluster membership probabilities of samples were then determined using the estimated component weights and parameters of the Gaussian components for this selected run. Cluster memberships of samples were then obtained in a hard/soft fashion using a maximum probability criterion. The robustness of the procedure was tested by performing ten separate 100 optimisation runs and comparing the best runs for each batch. R-code, *vabayelMix*, which implements the variational bayesian clustering algorithm is availablefrom the R-website [52]For the hierarchical clustering we used the R-function *hclust *using an euclidean distance metric.

### Independent component analysis

ICA [reviewed in [33]] was used here merely as an unsupervised projection pursuit algorithm to find one dimensional projections of the gene expression matrix *VG *that are multi-modal in expression space. These multi-modal projections are interesting since they may differentiate tissuetypes. Since a multi-modal projection is necessarily non-gaussian, a set ofsuch interesting projections or modes can be found by requiring these to bestatistically independent across sample space. In detail, the model used is (VG)st=∑lSslAlt
 MathType@MTEF@5@5@+=feaafiart1ev1aaatCvAUfKttLearuWrP9MDH5MBPbIqV92AaeXatLxBI9gBaebbnrfifHhDYfgasaacH8akY=wiFfYdH8Gipec8Eeeu0xXdbba9frFj0=OqFfea0dXdd9vqai=hGuQ8kuc9pgc9s8qqaq=dirpe0xb9q8qiLsFr0=vr0=vr0dc8meaabaqaciaacaGaaeqabaqabeGadaaakeaadaqadaqaaiabdAfawjabdEeahbGaayjkaiaawMcaamaaBaaaleaacqWGZbWCcqWG0baDaeqaaOGaeyypa0ZaaabuaeaacqWGtbWudaWgaaWcbaGaem4CamNaemiBaWgabeaakiabdgeabnaaBaaaleaacqWGSbaBcqWG0baDaeqaaaqaaiabdYgaSbqab0GaeyyeIuoaaaa@4054@, where the summation is over the independent modes *l*, and where *S *and *A *denote the "source" and "mixing" matrices respectively. Associated with each mode we have two variational patterns, one across genes (rows of *A*) and another across samples (columns of *S*). The columns of *S *are inferred using the criterion of statistical independence [33]. The estimation and uniqueness of the independent modes relies on the distribution of expression values of samples along these components being non-gaussian [33]. This requires a dimensional reduction to a maximally varying gene subspace to remove any gaussian noise components. A PCA (principal component analysis) was done to project the data onto such a maximally varying subspace. On our data set we found that a projection onto a seven-dimensional subspace was necessary to ensure the uniqueness of the modes. Inference was then carried out within a maximum likelihood framework (R-code, *mlica*, is available from [52]) usingan iterative procedure similar to the one suggested by Hyvaerinnen [33]. Robustness of the optimisation procedure to the initialisation point was ensured by performing 100 runs and selecting the run that maximised the log-likelihood. We further checked our estimated modes against an alternative implementation of ICA [*fastICA *53] that uses negative entropy as a non-gaussianity measure to estimate the mixing matrix. When reduced to the seven dimensional subspace determined by PCA we found complete consistency between the modes obtained via both methods. Consistent modes were sorted according to their relative data power [54].

### Classification and validation analysis

Our classification problem involved a relatively large number of categories(tissue types) and a small number of predictor variables (genes). Such a setting is well suited for a parametric mixture model approach [42,43,55]. Following [43] we performed the classification analysis using a Gaussian mixture model adapting the variational Bayesian algorithm for learning fromthe training set. We used two methods of internal cross-validation. In the leave-one-out method the test set was made up of a randomly selected samplefrom each tissue category. In the second method we randomly selected 20% of samples from each tissue category and placed them in the test set. Given a partition of the samples into a training and test set we applied the variational Bayesian Gaussian mixture model to learn from the training set the cluster means and variances for each tissue category. The learning was done for each tissue separately by setting K=1 in the model fitting. Test samples were then assigned to categories using the linear discriminant classifier [56]

*D*(*c *|*x*_*s*_) = - (*x*_*s *_- *μ*_*c*_)^*T*^Ω_*c*_(*x*_*s *_- *μ*_*c*_) + log(detΩ_*c*_) + 2log *w*_*c*_

where *c *labels the tissue type, *x*_*s *_is the expression vector of test sample *s*, *μ*_*c *_is the mean expression vector for category *c*, Ω_*c *_is the inverse covariance matrix (positive definite) for category *c *and *w*_*c *_is the prior weight for category *c*. We recorded the error rates on the training and test sets for 1000 different randomly selected partitions and for each of the two partitioning methods. The average and standard deviation of the test error rate over these 1000 random partitions were then computed. Finally,these statistics were computed for all possible combinations of genes allowing us to find the optimal classifier(s).

## Authors' contributions

**HÖ **participated in the design of the study, did the mutation analysis and QRT-PCR analysis, involved in the interpretation of the data, drafted the manuscript. **AET **did the statistical analysis, participated in the interpretation of the data, involved in drafting the manuscript. **AAA **did part of the statistical analysis, participatedin the interpretation of the data, involved in drafting the manuscript. **SJH **did the mutation analysis and QRT-PCR analysis. **CB **did QRT-PCR analysis. **LB **responsible for review of pathology of all breast samples. **AV **did QRT-PCR analysis. **GB **did QRT-PCR analysis. **TS **prepared ovarian tumour RNA and DNA samples. **MJA **responsible for review of pathology of all colorectal samples.**VPC **responsible for review of pathology of all glioma samples and provider of the respective RNAs. **DB **provided and contributed to the analysis of expression microarray dataset. **TK **involved in conception of study and drafting/revising the manuscript. **JDB **contributed to study design, contributed to the interpretation of the data;involved in drafting and revising the manuscript. **CC **conceived thestudy, involved in its design, analysis and interpretation, involved in drafting the manuscript and responsible for final manuscript editing.

## Supplementary Material

Additional File 1 Table S1; VG matrix for cell lines. Click here for file

Additional File 2 Table S2; VG matrix for primary tumours. Click here for file

Additional File 3 Hierarchical clustering; Hierarchical clustering of expression matrix across primary samples using all 12 genes. Red denotes overexpression, green underexpression. See Figure 2 for detailed expression values. Click here for file

Additional File 4 Independent component analysis (ICA); a. The projected sample expression data along directions identified through ICA. b. The associated gene weight vectors specifying the modes (directions) in gene space. The y-axis in both panels measures the relative activation level of the mode across samples and genes, respectively. The scales within each panel can be set arbitrarily since it is only the scale and sign of the product SA that indicates for each mode whether a gene is underexpressed oroverexpressed. For example, for projection 2 (ML-IC2) PCAF is overexpressedin renals relative to colorectal tumours. The scales in panel A were set sothat the columns of S have unit variance. c. Ensemble learning clustering on primary samples based on expression ratio of 4 chromatin remodelling genes. Red denotes overexpression, green underexpression. See Figure 2 for detailed expression values. Sample colour codes: as in Figure 2. Click here for file

Additional File 5 Table S3; For each pairwise comparison of cancer tissue types (breast, renal, colorectal and ovary) profiled in our study ♦ and in an independent microarray study ♠ we indicate the genes thatdiscriminated the two tissue types according to the Wilcoxon rank sum test (p < 0.01). NP means not profiled in microarray study. Last row gives the error rates obtained on test set using 20% internal cross validation on the microarray data and the genes marked ♠ in the classifier. Click here for file

Additional File 6 Table S4; List of cell lines. Click here for file

Additional File 7 Table S5; Primer sequences used in mutation analysis. Click here for file

Additional File 8 Table S6; Primer sequences used in real time PCR. Click here for file

Additional File 9 F statistics and the normalisation approach. Click here for file

## Figures and Tables

**Table 1:** The range, mean value and variance of expression of the target genes across all samples.

	**Primary samples**				**Cell Lines**			
**GENE**	**Minimum**	**Maximum**	**Mean**	**Variance**	**Minimum**	**Maximum**	**Mean**	**Variance**
**HDAC1**	-10	5.4	1.5	2.1	-1.9	2.5	1	1.2
**HDAC2**	-4.6	6.3	1.3	1.1	-2.1	3.2	1.8	1.9
**HDAC4**	-6.6	5.9	2.7	1.5	-6.1	3.8	4.2	2.1
**HDAC5**	-6.5	6.5	1.7	2.1	-7.2	5.9	0.2	3.8
**HDAC7A**	-3.8	3.1	4.6	1.7	-2.7	3.9	4.7	1.5
**SIRT1**	-3.7	5.8	-0.2	4.8	-4.9	3.4	-0.9	6.7
**SUV39H1**	-3	3.9	0.9	1.2	-1.3	3	1.7	1.2
**SUV39H2**	-1.4	6.7	1	1.6	-2.8	3.2	1.5	1.5
**EZH2**	-4.9	7.2	0.5	3.2	-4.2	5.1	0.4	2.9
**CREBBP**	-3.3	8.1	-0.5	6.4	-2.1	4.9	2.5	2.6
**P300**	-4.5	4.8	1.3	1.1	-1.5	2.9	0.7	0.9
**PCAF**	-7.5	4.5	2.7	2.9	-3.8	4.3	-0.2	3.2

**Table 2:** Differential expression analysis of tumour-normal pairs using a Wilcoxon rank sum test at a 0.001 significance level.

	**Breast Tum-Nor**	**Renal Tum-Nor**	**Bladder Tum-Nor**	**Colorectal Tum-Nor**
**SUV39H2**	0	0	0	0
**SUV39H1**	0	1	0	100
**SIRT1**	0	1	0	100
**PCAF**	0	37	28	0
**P300**	0	0	0	0
**HDAC7A**	0	0	0	100
**HDAC5**	6	0	0	100
**HDAC4**	97	0	0	0
**HDAC2**	0	0	0	0
**HDAC1**	0	0	0	100
**EZH2**	81	100	0	0
**CREBBP**	100	0	0	0

Rows label target genes, columns label tissue types. Numbers in table represent a robustness measure of the differential expression between the tumour-normal pair: they equal the number of times (out of 100 bootstrapped data and expression estimate sets) that the differential expression was significant at the 0.001 level.

**Table 3:** Distribution of tumour and normal samples into clusters based on the normalized expression ratio of 12 chromatin remodelling genes.

	**BrTum**	**BrNor**	**RenT**	**RenN**	**BITum**	**BINor**	**CrTum**	**CrNor**	**Gilo**	**Ovarian**
Chapter1	92%	100%				17%				
Chapter2	4%				6%					23%
Chapter3	4%				6%				100%	73%
Chapter4			100%	100%	88%	83%				
Chapter5							100%	100%		2%
Chapter6										2%

**Table 4:** Mean and standard deviation of the error on test and training sets obtained in internal cross-validation using a 20% test set. Error rates shown only for the optimal classifiers for each possible number of genes in the classifier.

**Classifier**	**Mean error ± std (Train. Set)**	**Mean error ± (Test Set)**
***SIRT1 & REBBP***	0.24 ± 0.02	0.26 ± 0.05
*SIRT1, CREBBP & HDAC7A*	0.17 ± 0.02	0.20 ± 0.05
*SIRT1, CREBBP, HDAC7A & HDAC5*	0.15 ± 0.02	0.16 ± 0.05
*SIRT1, CREBBP, HDAC7A, HDAC5 & PCAF*	0.13 ± 0.02	0.14 ± 0.05
*SIRT1, CREBBP, HDAC7A, HDAC5, PCAF & EZH2*	0.13 ± 0.02	0.15 ± 0.05
*SIRT1, CREBBP, HDAC7A, HDAC5, PCAF, EZH2 & SUV39H1*	0.12 ± 0.02	0.15 ± 0.05

**Table 5:** Summary of mutations identified in *SUV39H1, SUV39H2*, and *HDAC2*.

**Gene**	Sample	**Mutation**	**Codon**
*SUV39H1*	UCI101	862C>T	Q288STOP
*HDAC2*	HCT15	1637DelA	FS541

FS: Frameshift

**Table 6:** Summary of other sequence alterations identified in *SUV39H1, SUV39H2*, and *HDAC2*

**Gene**	Sample	**Frequency**	**Sequence Alteration**	**Codon**
SUV39H1	4 Ov. Tum.	2%	IVS2-69G>C	
	1 Ov. Tum.	0.5%	525C>T	F260F
SUV39H2	1 Br. Tum.	0.5%	55insT(5'UTR)	
	4CR, 1 Ov, 1 Br.Tumour, 1 Ov. Cell Line	(4%)	442A>C	R74Q
	14 Ca.CL., 7 CR, 12Br, 12 Ov. Tum.	20%	1008C>T	Y336Y
	3 Br., 1 Ov. Tum.	2%.	1083C>G	L361L
HDAC1	1 Br. Tum	0.5%	1212G>A	A383A
HDAC2	10 Ca. CL, 14 Br, 7 Ov, 3 CR Tum.	18%	143InsCAG (5'UTR)	
	19 Ca. CL., 4 CR, 29 Br, 7 Ov. Tum.	32%	IVS4+30T>A	
	2 Ov., 1 Br. Tum.	1.6%.	IVS4-9C>A	
	15 Ca. CL., 5 CR, 4 Br, 1 Ov. Tum.	14%	IVS11-13A>G	
	1 Ov. Ca. CL., Lymphocyte	1.1%	IVS13-26A>T	

Br. Breast, Ov. Ovarian, CR. Colorectal

## References

[B1] Lund AH, van Lohuizen M (2004). Epigenetics and cancer. Genes and Dev.

[B2] Santos-Rosa H, Caldas C (2005). Chromatin modifier enzymes, the histone code and cancer. Eur J Can.

[B3] Khorasanizadeh S (2004). The nucleosome: From genomic organization to genomic regulation. Cell.

[B4] Jenuwein T, Allis CD (2001). Translating the histone code. Science.

[B5] Berger SL (2002). Histone modifications in transcriptional regulation. Curr Opin Genet Dev.

[B6] Suzuki H, Gabrielson E, Chen W, Anbazhagan R, van Engeland M, Weijenberg MP, Herman JG, Baylin SB (2002). A genomic screen for genes upregulated by demethylation and histone deacetylase inhibition in human colorectal cancer. Nat Genet.

[B7] Glaser KB, Staver MJ, Waring JF, Stender J, Ulrich RG, Davidsen SK (2003). Gene expression profiling of multiple histone deacetylase (HDAC) inhibitors: defining a common gene set produced by HDAC inhibition in T24and MDA carcinoma cell lines. Mol Cancer Ther.

[B8] Sterner DE, Berger SL (2000). Acetylation of histones and transcription-related factors. Microbiol Mol Biol Rev.

[B9] Wolf D, Rodova M, Miska EA, Calvet JP, Kouzarides T (2002). Acetylation of beta-catenin by CREB-binding protein (CBP). J Biol Chem.

[B10] Thiagalingam S, Cheng KH, Lee HJ, Mineva N, Thiagalingam A, Ponte JF (2003). Histone deacetylases: unique players in shaping the epigenetic histone code. Ann N Y Acad Sci.

[B11] Schneider R, Bannister AJ, Myers FA, Thorne AW, Crane-Robinson C, Kouzarides T (2004). Histone H3 lysine 4 methylation patterns in highereukaryotic genes. Nat Cell Biol.

[B12] Cao R, Wang L, Wang H, Xia L, Erdjument-Bromage H, Tempst P, Jones RS, Zhang Y (2002). Role of histone H3 lysine 27 methylation in Polycomb-group silencing. Science.

[B13] Bannister AJ, Schneider R, Kouzarides T (2002). Histonemethylation: dynamic or static?. Cell.

[B14] Futreal PA, Coin L, Marshall M, Down T, Hubbard T, Wooster R, Rahman N, Stratton MR (2004). A census of human cancer genes. NatRev Cancer.

[B15] Muraoka M, Konishi M, Kikuchi-Yanoshita R, Tanaka K, Shitara N, Chong JM, Iwama T, Miyaki M (1996). p300 gene alterations in colorectal and gastric carcinomas. Oncogene.

[B16] Gayther SA, Batley SJ, Linger L, Bannister A, Thorpe K, Chin SF, Daigo Y, Russell P, Wilson A, Sowter HM, Delhanty JD, Ponder BA, Kouzarides T, Caldas C (2000). Mutations truncating the EP300 acetylase inhuman cancers. Nat Genet.

[B17] Choi JH, Kwon HJ, Yoon BI, Kim JH, Han SU, Joo HJ, Kim DY (2001). Expression profile of histone deacetylase 1 in gastric cancer tissues. Jpn J Cancer Res.

[B18] Kawai H, Li H, Avraham S, Jiang S, Avraham HK (2003). Overexpression of histone deacetylase HDAC1 modulates breast cancer progression by negative regulation of estrogen receptor alpha. Int J Cancer.

[B19] Vaziri H, Dessain SK, Ng Eaton E, Imai SI, Frye RA, Pandita TK, Guarente L, Weinberg RA (2001). hSIR2 (SIRT1) functions as an NAD-dependent p53 deacetylase. Cell.

[B20] Cheng HL, Mostoslavsky R, Saito S, Manis JP, Gu Y, Patel P, Bronson R, Appella E, Alt FW, Chua KF (2003). Developmental defects and p53 hyperacetylation in Sir2 homolog (SIRT1)-deficient mice. Proc Natl Acad Sci U S A.

[B21] Peters AH, O'Carroll D, Scherthan H, Mechtler K, Sauer S, Schofer C, Weipoltshammer K, Pagani M, Lachner M, Kohlmaier A, Opravil S, Doyle M, Sibilia M, Jenuwein T (2001). Loss of the Suv39h histone methyltransferases impairs mammalian heterochromatin and genome stability. Cell.

[B22] Nielsen SJ, Schneider R, Bauer UM, Bannister AJ, Morrison A, O'Carroll D, Firestein R, Cleary M, Jenuwein T, Herrera RE, Kouzarides T (2001). Rb targets histone H3 methylation and HP1 to promoters. Nature.

[B23] Varambally S, Dhanasekaran SM, Zhou M, Barrette TR, Kumar-Sinha C, Sanda MG, Ghosh D, Pienta KJ, Sewalt RG, Otte AP, Rubin MA, Chinnaiyan AM (2002). The polycomb group protein EZH2 is involved in progression of prostate cancer. Nature.

[B24] Kleer CG, Cao Q, Varambally S, Shen R, Ota I, Tomlins SA, Ghosh D, Sewalt RG, Otte AP, Hayes DF, Sabel MS, Livant D, Weiss SJ, Rubin MA, Chinnaiyan AM (2003). EZH2 is a marker of aggressive breast cancer and promotes neoplastic transformation of breast epithelial cells. Proc Natl Acad Sci U S A.

[B25] Paz MF, Fraga MF, Avila S, Guo M, Pollan M, Herman JG, Esteller M (2003). A systematic profile of DNA methylation in human cancer cell lines. Can Res.

[B26] Fraga MF, Ballestar E, Villar-Garea A, Boix-Chornet M, Espada J, Schotta G, Bonaldi T, Haydon C, Propero S, Petrie K, Iyer NG, Perez-Rosado A, Calvo E, Loper JA, Cano A, Calasanz MJ, Colomer D, Piris MA, Ahn N, Imhof A, Caldas C, Jenuwein T, Esteller M (2005). Loss of acetylation at Lys16 and trimethylation at Lys20 of histone H4 is a common hallmark of human cancer. Nat Genet.

[B27] Seligson DB, Horvath S, Shi T, Yu H, Tze S, Grunstein M, Kurdistani SK (2005). Global histone modification patterns predict risk ofprostate cancer recurrence. Nature.

[B28] Pfaffl M (2001). A new mathematical model for relative quantification in real-time RT-PCR. Nucleic Acids Res.

[B29] Vandesompele J, De Preter K, Pattyn F, Poppe B, Van Roy N, De Paepe A, Speleman F (2002). Accurate normalization of real-time quantitative RT-PCR data by geometric averaging of multiple internalcontrol genes. Genome Biology.

[B30] Livak K, Schmittgen T (2001). Analysis of Relative Gene Expression Data using Real-Time Quantitative PCR. Methods.

[B31] Wu CF (1986). Jackknife, Bootstrap and Other Resampling Methods in Regression Analysis. The Annals of Statistics.

[B32] Efron B (1979). Bootstrap Methods: Another Look at the Jackknife. The Annals of Statistics.

[B33] Hyvaerinnen A, Karhunen J, Oja E (2001). Independent Component Analysis. Wiley.

[B34] Eisen M, Spellman P, Brown P, Botstein D (1998). Cluster analysis and display of genome-wide expression patterns. Proc Natl Acad Sci USA.

[B35] Tavazoie S, Hughes J, Campbell M, Cho R, Church G (1999). Systematic determination of genetic network architecture. Nat Genet.

[B36] Ghosh D, Chinnaiyan A (2002). Mixture modelling of gene expression data from microarray experiments. Bioinformatics.

[B37] MacKay D (1995). Developments in probabilistic modelling with neural networks-ensemble learning. In Neural Networks: Artificial Intelligence and Industrial Applications. Proceedings of the 3rd Annual Symposium on Neural Networks.

[B38] Teschendorff A, Wang Y, Barbosa-Morais L, Brenton J, Caldas C (2005). A variational Bayesian mixture modelling framework for cluster analysis of gene expression data. Bioinformatics.

[B39] McShane L, Radmacher M, Freidlin B, Yu R, Li MC, Simon R (2002). Methods for assessing reproducibility of clustering patterns observed in analyses of microarray data. Bioinformatics.

[B40] van de Vijver MJ, He YD, van't Veer LJ, Dai H, Hart AA, Voskuil DW, Schreiber GJ, Peterse JL, Roberts C, Marton MJ, Parrish M, Atsma D, Witteveen A, Glas A, Delahaye L, van der Velde T, Bartelink H, Rodenhuis S, Rutgers ET, Friend SH, Bernards R (2002). A gene-expression signature as a predictor of survival in breast cancer. N Engl J Med.

[B41] Alizadeh AA, Eisen MB, Davis RE, Ma C, Lossos IS, Rosenwald A, Boldrick JC, Sabet H, Tran T, Yu X, Powell JI, Yang L, Marti GE, Moore T, Hudson J, Lu L, Lewis DB, Tibshirani R, Sherlock G, Chan WC, Greiner TC, Weisenburger DD, Armitage JO, Warnke R, Levy R, Wilson W, Grever MR, Byrd JC, Botstein D, Brown PO, Staudt LM (2000). Distinct types of diffuse large B-cell lymphoma identified by gene expression profiling. Nature.

[B42] Dudoit S, Fridlyand J, Speed T (2002). Comparison of Discrimination Methods for the Classification of Tumors Using Gene Expression Data. Journal of the American Statistical Association.

[B43] Fraley C, Raftery AE (2002). Model-based clustering, discriminant analysis and density estimation. Journal of the American Statistical Association.

[B44] Tothill RW, Kowalczyk A, Rischin D, Bousioutas A, Haviv I, van Laar RK, Waring PM, Zalcber J, Ward R, Biankin AV, Sutherland RL, Henshall SM, Fong K, Pollack JR, Bowtell DDL, Holloway AJ (2005). An expression-based site of origin diagnostic method designed for clinical application to cancer of unknown origin. Can Res.

[B45] Özdağ H, Batley SJ, Forsti A, Iyer NG, Daigo Y, Boutell J, Arends MJ, Ponder BA, Kouzarides T, Caldas C (2002). Mutation analysis of CBP and PCAF reveals rare inactivating mutations in cancer cell lines but not in primary tumours. Br J Cancer.

[B46] Kishimoto M, Kohno T, Okudela K, Otsuka A, Sasaki H, Tanabe C, Sakiyama T, Hirama C, Kitabayashi I, Minna JD, Takenoshita S, Yokota J (2005). Mutations and deletions of the CBP gene in human lung cancer. Clin Can Res.

[B47] Ward R, Johnson M, Shridhar V, Van Deursen J, Couch FJ (2005). CBP truncating mutations in ovarian cancer. J Med Genet.

[B48] Zhu P, Martin E, Mengwasser J, Schlag P, Janssen K-P, Göttlicher M (2004). Induction of HDAC2 expression upon loss of APC in colorectal tumorigenesis. Cancer Cell.

[B49] http://insertion.stanford.edu/melt.html.

[B50] Weir BS (1996). Genetic Data Analysis II. Wiley, Sinauer Associates.

[B51] R Development Core Team (2003). R: A language and environment for statistical computing. R Foundation for Statistical Computing.

[B52] http://www.cran.r-project.org.

[B53] Hyvaerinnen A (1999). Fast and Robust Fixed-Point Algorithms for Independent Component Analysis. IEEE Transactions on Neural Networks.

[B54] Kreil DP, MacKay DJC (2003). Reproducibility Assessment of Independent Component Analysis of Expression Ratios from DNA microarrays. Comparative and Functional Genomics.

[B55] Baldi P (2001). Bioinformatics: the machine learning approach.

[B56] Dudoit S, Fridlyand J, Speed T (2002). Comparison of Discrimination Methods for the Classification of Tumors Using Gene Expression Data. Journal of the American Statistical Association.

