# Supplementary material for: Differential expression of selected histone modifier genes in human solid cancers
Source: BMC Genomics. 2006 Apr 25;7:90. doi: 10.1186/1471-2164-7-90 (PMC1475574; doi:10.1186/1471-2164-7-90)
Supplement: Additional File 5 — Table S3; For each pairwise comparison of cancer tissue types (breast, renal, colorectal and ovary) profiled in our study ♦ and in an independent microarray study ♠ we indicate the genes thatdiscriminated the two tissue types according to the Wilcoxon rank sum test (p < 0.01). NP means not profiled in microarray study. Last row gives the error rates obtained on test set using 20% internal cross validation on the microarray data and the genes marked ♠ in the classifier. [file 1471-2164-7-90-S5.doc]

| *Gene|Tissue type pair* | **Ovary- Colon** | **Ovary-Breast** | **Ovary-Renal** | **Colon-**  **Breast** | **Colon-**  **Renal** | **Breast-**  **Renal** |
| --- | --- | --- | --- | --- | --- | --- |
| *HDAC1* | ♣ ♦ | ♣ | ♣ ♦ | ♣ ♦ | ♣ | ♦ |
| *HDAC2* | ♦ |  | ♣ | ♦ | ♣ ♦ | ♦ |
| *HDAC4* | ♦ | ♣ | ♣ ♦ | ♦ | ♣ ♦ | ♦ |
| *HDAC5* | ♦ | ♦ | ♦ | ♦ | ♦ |  |
| *HDAC7A* | *NP* ♦ | *NP* | *NP* ♦ | *NP* ♦ | *NP* ♦ | *N*P ♦ |
| *SIRT1* | ♦ | ♦ | ♦ | ♦ | ♦ | ♦ |
| *SUV39H1* | ♦ |  | ♦ | ♦ | ♦ | ♦ |
| *SUV39H2* | *NP* ♦ | *NP* ♦ | *NP* | *NP* | *NP* | *NP* ♦ |
| *EZH2* | ♦ | ♦ |  | ♣ ♦ | ♣ ♦ | ♣ ♦ |
| *CREBBP* | ♦ | ♦ | ♦ | ♣ ♦ |  | ♣ ♦ |
| *EP300* | ♣ | ♦ | ♦ | ♣ ♦ | ♦ | ♦ |
| *PCAF* |  | ♦ | ♦ | ♦ | ♦ | ♦ |
| *Error rate ♣* | 0.29±0.11 | 0.27±0.10 | 0.10±0.08 | 0.15±0.10 | 0.06±0.08 | 0.16±0.12 |
