# Supplementary material for: Differential expression of selected histone modifier genes in human solid cancers
Source: BMC Genomics. 2006 Apr 25;7:90. doi: 10.1186/1471-2164-7-90 (PMC1475574; doi:10.1186/1471-2164-7-90)
Supplement: Additional File 9 — F statistics and the normalisation approach. [file 1471-2164-7-90-S9.pdf]

Supplementary Information

1. Gene stability measures

Since the expression stability of a candidate reference gene can not be judged without reference to another gene it was proposed in [1] to use a gene stability measure based on pairwise comparisons of candidate reference genes. Here we propose a similar measure but use efficiency corrected Ct values [2] instead of just Ct values. This is important since otherwise we are confounding the variability of expression with gene variations in amplification efficiency and sample loading differences [2, 3].

To motivate the gene stability measure we are about to propose we start with the log-transform of the expression ratio between sample and control as given in [2],

$$\begin{aligned} y_{tsrc} &= \log_2 R_{tsrc} \\ &= \log_2 \frac{E_t^{(Ct)_{tc}-(Ct)_{ts}}}{E_r^{(Ct)_{rc}-(Ct)_{rs}}} \end{aligned} \quad (1)$$

where r denotes the reference gene, t the target gene, s the sample, c the control sample, E_g the efficency of gene g (which can be either the target gene t or the reference gene r) and $(Ct)_{gx}$ the measured Ct value for gene g in sample x. This formula is derived from an exponential model for PCR amplification. Because it is model-based we consider it to be a robust measure to use for relative quantification of RT-PCR data. However, in deriving (1) several assumptions were made. One important assumption is that the transcipt level of the reference gene used is the same between sample and control. A second minor assumption is that the amplification efficiency only depends on the gene and is therefore sample independent.

Since the aim is to carry out inferences from (1) we can gauge the error incurred by assuming stable housekeeping gene expression by considering the pairwise expression ratios $y_{r'src}$. We assume that a set of n_r candidate reference genes has been selected using prior knowledge gained from other RT-PCR or microarray experiments [4]. Clearly, these candidate control genes are chosen with the prior knowledge that they show little expression variability between the samples in the study. If the selected housekeeping genes are true housekeepers and if we ignore other minor sources of error, then the variability of the pairwise ratios $y_{r'src}$ across samples would be zero.

Generally, we are interested in infering differential expression between types of samples within a sample set of size N_s. In what follows, let C denote the number of different types or categories, c label the category, $(cj) = s$ denote a sample within category c and N_c denote the number of samples within category c. Then, for any gene g, be it a target gene t or candidate reference gene r, we can write the total variation (sum of squares) as measured using a reference gene r' as a sum of a between-types and a within-types component,

$$\begin{aligned} SS_{gr'} &\equiv \sum_{c,j}(y_{gcjr'} - y_{g\cdot\cdot r'})^2 \\ &= \sum_{cj}(y_{gcjr'} - y_{gc\cdot r'})^2 + \sum_{c} N_c(y_{gc\cdot r} - y_{g\cdot\cdot r'})^2 \\ &= SS_{gr'}^{(W)} + SS_{gr'}^{(B)} \end{aligned} \quad (2)$$

where $\cdot$ denotes the mean over that index. Expressed in terms of the variance and the between and within mean square components equation (2) is

$$V_{gr'} = \frac{N_s - C}{N_s - 1} MS_{gr'}^{(W)} + \frac{C-1}{N_s-1} MS_{gr'}^{(B)} \quad (3)$$

Since we have a set of candidate reference genes we can define an overall noise level for reference gene r, N_r, by taking the average over all the other reference genes, i.e,

$$N_r \equiv \frac{1}{n_r - 1} \sum_{r' \neq r} V_{rr'} \quad (4)$$

Similarly, one may define an overall signal level for target gene t, S_t, by

$$S_t \equiv \frac{1}{n_r} \sum_{r'} V_{tr'} \quad (5)$$

Other stability measures that quantify the signal and noise levels within types as well as between types can be obtained by taking the averages of $(MS)^{(W)}$ and $(MS)^{(B)}$, respectively.

The variance measures $V_{tr'}$ and $V_{rr'}$ for our RT-PCR study of 178 samples (comprising 27 breast tumours, 5 breast normals, 12 renal tumour/normal pairs, 18 bladder tumours, 6 bladder normals, 20 colorectal tumour/normal pairs, 15 glioblastomas and 45 ovarian cancers), 12 histone modifier genes *HDAC1, HDAC2, HDAC5, HDAC7A, SIRT1, SUV39H1,*

SUV39H2, EZH2, CREBBP, EP300 and PCAF and 8 candidate reference genes *ACTB, B2M, GAPDH, HMBS, HPRT, RPL3, SDH and UBC* are shown in Figures 1,2 & 3. It is clear from these Figures that without further analysis reliable inferences are only possible for approximately 7 target genes. Thus, to ensure that inferences for all 12 target genes are reliable, careful quantification of the noise is paramount.

2. The signal to noise ratio F-statistic

The advantage of the stability measures as defined here is that it allows direct comparison to the magnitude of the target gene signals. Clearly, to make reliable inferences about a target gene we require that it shows more variability across samples than the housekeeping genes themselves, otherwise no inferences can be made. This is tantamount to requiring that the signal to noise ratio (SNR) be larger than a threshold value of 1.

Since the noise (or error) introduced depends on the housekeeping gene used we define a SNR for each target-reference gene pair. To estimate the signal and noise levels we propose to use the stability measures defined previously. Thus, for each target-reference gene we propose the following F-statistic

$$F_{tr} \equiv \frac{S_t}{N_r} = \frac{n_r - 1}{n_r} \frac{\sum_{r'} V_{tr'}}{\sum_{r \neq r'} V_{rr'}} \qquad (6)$$

where $V_{tr'}$ and $V_{rr'}$ are given by equation (3). If this statistic is larger than 1 we would consider the total signal level of target gene t to be above the noise level introduced by reference gene r. Since in general we may also seek comparisons between tissue types we would also require that the between-type signal level be larger than the between-type noise level. Thus, a similar statistic, $F^{(B)}_{tr}$ would be required that is defined through equation (6) but with V replaced by $(MS)^{(B)}$.

The distribution of the statistic (6) is unknown. We can however obtain an approximation to this distribution by bootstrapping over the reference gene set r' in the numerator and denomenator separately, provided we have a sufficient number of candidate reference genes. Given a sample set of values of some statistic whose distribution is unknown we can obtain an approximation to it by fitting a density function to a histogram of a large number of bootstrap means, see e.g [5]. The fundamental assumption underlying the bootstrap idea is that the sample set of values is representative of a much larger population of values. For the sample set to be representative requires a sufficient number of samples values, in practice this means more than 6. For the case at hand the assumption requires the candidate reference gene set to form a reasonably good representation of a much larger population of genes that don't show much variability across the samples. By bootstrapping over the candidate set we obtain an approximation to a distribution that models noise due to unstable housekeeping gene expression.

3. The bootstrap variance test

Using the bootstrap idea we now propose the following statistical procedure to decide whether a given candidate control gene can be used to make inferences about a target genes' expression variability:

1. Initialise a counter to zero.
2. Bootstrap over reference genes in the numerator and denomenator of (6) separately.
3. Compute the resulting statistic $(SNR)^{(b)}_{tr}$ and add 1 to the counter if $(SNR)^{(b)}_{tr} < 1$.
4. Repeat the previous two steps a large number of times N_b (~ 1000).
5. We interpret the fraction of times the $(SNR)^{(b)}_{tr}$ was larger than 1 as a p-value for rejecting the null hypothesis that the target gene does not show more variability than the reference gene.

4. Selection of valid housekeepers

Application of this statistical test will result in a set of suitable housekeeping genes for making inferences about a target gene. Similarly, the test may identify reference genes that are not suitable as housekeepers because they are not stably expressed relative to a large proportion of the target genes in the study,in which case they can be removed from further analysis. The number of housekeeping genes to use in downstream analysis not only depends on the results of the F-test, but also on the specific application. Generally, the number of housekeepers to use has to be traded against the minimum number of target genes that one wishes to make reliable inferences on (Tables 1 & 2). Combining the results of

the statistical test for the total and between pairwise measures, we can see that for example inferences on up to eight target genes (all except *HDAC1, HDAC2, HDAC4 and EP300*) are possible provided we use as housekeeping genes *SDH, HMBS, HPRT, RPL3 and UBC*. Alternatively, if inferences are restricted to the five target genes *CREBBP, SIRT1, HDAC7A, EZH2 and SUV39H1* we can use all eight housekeeping genes. We decided in our study to use as housekeepers all the reference genes that were stable relative to at least 5 of the target genes. Using this approach we would expect that robust inferences can be made for at least these 5 target genes. While it may seem that this may compromise possible inferences for the other target genes, the robustness of these inferences can always be evaluated using the linear model approach proposed next.

5. An analysis of variance model for normalisation and quantification of normalisation errors

Having selected a subset of housekeeping genes, we next suggest a model-based approach for subsequent normalisation and quantification of the normalisation errors incurred. The error introduced by non-ideal houskeeping gene conditions can be quantified by fitting a fixed effects linear model to the log-ratios,

$$\begin{aligned} y^{(i)}_{tsre} &= \mu + G_t + V_s + R_r \\ &\quad + (VG)_{st} + (VR)_{sr} + \epsilon^{(i)}_{tsre} \end{aligned} \quad (7)$$

where $t = 1...n_t$ denotes the target gene, $r = 1...n'_r$ denotes the housekeeping gene, $s = 1...N_s$ denotes the sample,e denotes an additional efficiency measurement (obtained in a different sample than the control), and $i = 1...N_R$ denotes replicate measurements. In general, we may not have many independent replicate measurements since Ct values are usually measured only in triplicate. However, it is possible to combine the triplicates in the log-ratios using a bootstrapping procedure to generate a significant number $N_R \sim 10$ of replicates. In the above G_t, V_s, and R_r stand for the gene, sample and reference singleton effects. Thus, $\mu + R_r$ is the average expression of all target genes across all samples relative to the control sample when measured using housekeeping gene r. The other fixed terms include the interactions between the various factors. We have not included a target-housekeeping gene interaction $(GR)_{tr}$ since it turns out that this interaction is not statistically significant. Similarly, the multiple efficiency measurements available could be incorporated into the model. While inclusion of such terms yielded a significant p-value their effect is small compared with the signals due to target and reference genes and therefore are incorporated as a random effect into $\epsilon^{(i)}_{tsre}$, which we assume is a gaussian of mean zero and variance σ^2_t.

The parameters are estimated using maximum likelihood. This amounts to simple averaging over factors. The normalised expression matrix $(\overline{VG})_{st}$ is thus obtained as

$$(\overline{VG})_{st} \equiv \hat{\mu} + \hat{V}_s + \hat{G}_t + (\widehat{VG})_{st} \quad (8)$$

where ˆ denotes the estimate for the parameter.

Quantification of normalisation errors can be done through an analysis of variance [] of the model (7). The effect of the normalisation error on the estimated expression matrix $\overline{VG}$ can be modelled by a bootstrap approach similar to the one suggested in [6] and [7]. Explicitly, we first obtain perturbations of $\overline{VG}$ that represent the error arising from non-ideal housekeeping gene conditions by fitting to the expression ratios a modification of model (7) with $VR = 0$. The variance of VR is then captured by the estimated residuals $\epsilon^{(i)}_{tsre}$. We next bootstrap over the residuals for each target gene t separately. This randomises both samples and housekeeping genes. The bootstrapped residuals are then added to the parameter estimates and the modified model is refitted to yield a new matrix $\overline{VG}^{(b)}$ representing a perturbation around $\overline{VG}$. Finally, a large number of bootstraps is performed to obtain confidence intervals for each entry in the matrix $\overline{VG}$. Robust inferences about expression differences can then be made by consideration of these confidence intervals.

References

1. Vandesompele J, De Preter K, Pattyn F, Poppe B, Van Roy N, De Paepe A, Speleman F: **Accurate normalization of real-time quantitative RT-PCR data by geometric averaging of multiple internal control genes**. *Genome Biol* 2002, **3**(7):RESEARCH0034.
2. Pfaffl MW: **A new mathematical model for relative quantification in real-time RT-PCR**. *Nucleic Acids Res* 2001, **29**(9):e45.
3. Livak KJ, Schmittgen TD: **Analysis of relative gene expression data using real-time quantitative PCR and the 2(-Delta Delta C(T)) Method**. *Methods* 2001, **25**(4):402–408.

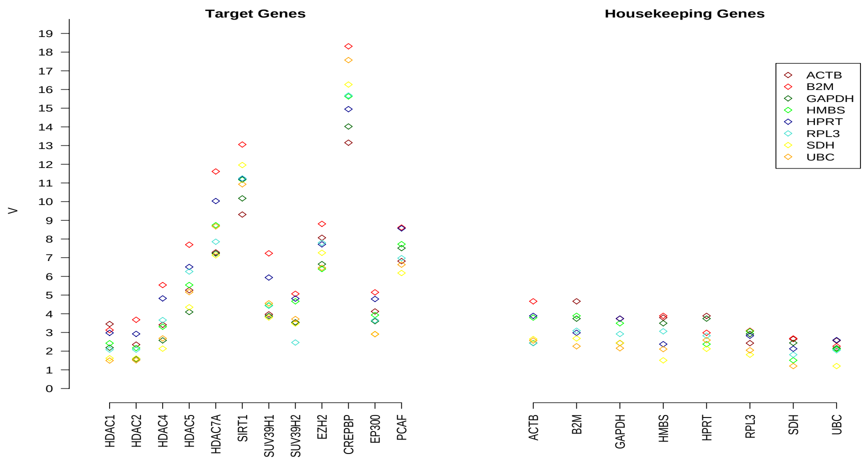

Figure 1: Total pairwise variance measures $V_{tr'}$ and $V_{rr'}$ over whole sample set.

4. Szabo A, Perou CM, Karaca M, Perreard L, Quackenbush JF, Bernard PS: **Statistical modeling for selecting housekeeper genes**. *Genome Biol* 2004, **5**(8):R59.
5. Weir B: *Genetic Data Analysis II*. Massachussets: Wiley, Sinauer Associates 1996.
6. Efron B: **Bootstrap Methods: Another Look at the Jackknife**. *The Annals of Statistics* 1979, **7**:1–26.
7. Wu CFJ: **Jackknife, Bootstrap and Other Resampling Methods in Regression Analysis**. *The Annals of Statistics* 1986, **14**(4):1261–1295.

Figures

Figure 1 - Total pairwise variance measures.

Total pairwise variance measures $V_{tr'}$ and $V_{rr'}$ over whole sample set.

Figure 2 - Between tissues pairwise variance measures.

Between tissues pairwise variance measures $V_{tr'}$ and $V_{rr'}$ over whole sample set.

Figure 3 - Within tissues pairwise variance measures.

Within tissues pairwise variance measures $V_{tr'}$ and $V_{rr'}$ over whole sample set.

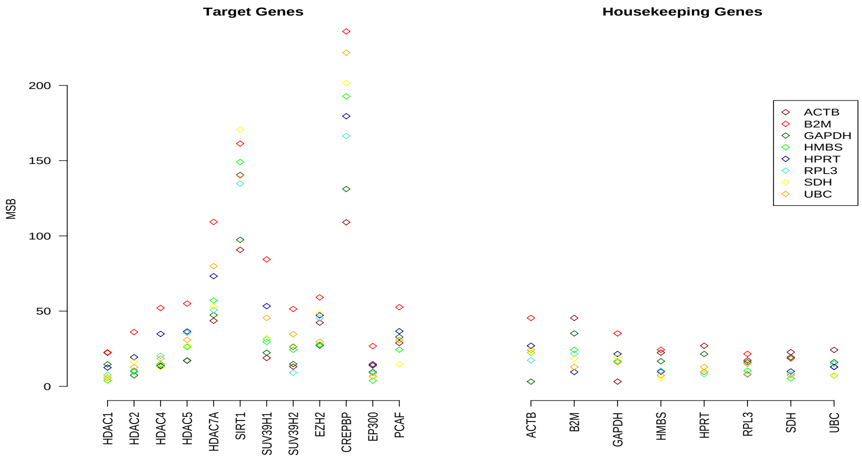

Figure 2: Between tissues pairwise variance measures $V_{tr'}$ and $V_{rr'}$ over whole sample set.

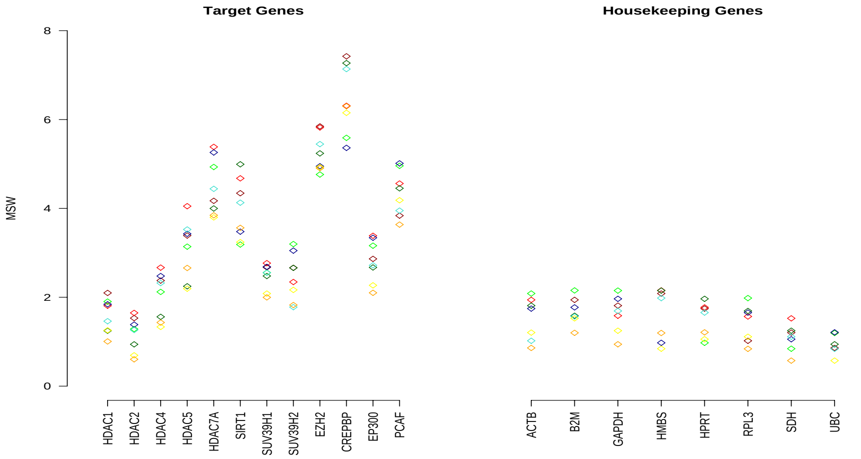

Figure 3: Within tissues pairwise variance measures $V_{tr'}$ and $V_{rr'}$ over whole sample set.

Tables

Table 1 - Target genes for inference and valid housekeeping gene subset. Target gene list is ranked according to best Signal-to-Noise ratio. Housekeeping gene list is ranked according to stability. Total pairwise measures.

Number	Inference for Target Genes & Stable Housekeeping Genes
5,6,7	*CREPBP, SIRT1, HDAC7A, EZH2, PCAF, HDAC5, SUV39H1*
	SDH, UBC, RPL3, HMBS, GAPDH, HPRT, ACTB, B2M
8	*CREPBP, SIRT1, HDAC7A, PCAF, EZH2, HDAC5, SUV39H1, EP300*
	SDH, UBC, RPL3, HMBS, GAPDH, ACTB, HPRT
9	*CREPBP, SIRT1, HDAC7A, PCAF, EZH2, HDAC5, SUV39H1, SUV39H2, EP300*
	SDH, UBC, RPL3, HMBS, HPRT, GAPDH
10	*CREPBP, SIRT1, HDAC7A, EZH2, PCAF, HDAC5, SUV39H1, SUV39H2, EP300, HDAC4*
	SDH, UBC, RPL3
11,12	*None*
	None

Table 2 - Target genes and valid housekeeping gene subset. Target gene list is ranked according to best Signal-to-Noise ratio. Housekeeping gene list is ranked according to stability. Between tissue pairwise measures.

Number	Inference for Target Genes & Stable Housekeeping Genes
5	*CREPBP, SIRT1, HDAC7A, EZH2, SUV39H1*
	SDH, UBC, HMBS, RPL3, HPRT, GAPDH, ACTB, B2M
6,7	*CREPBP, SIRT1, HDAC7A, EZH2, SUV39H1, HDAC5, PCAF*
	HMBS, SDH, RPL3, UBC, HPRT, GAPDH
8,9	*CREPBP, SIRT1, HDAC7A, EZH2, SUV39H1, HDAC5, PCAF, SUV39H2, HDAC4*
	SDH, HMBS, HPRT, RPL3, UBC
10,11,12	*None*
	None

Table 3 - Analysis of Variance of the linear model 7. For each factor, the degrees of freedom, mean square variation, F-statistic value and p-value are given.

Factor	dof	MS	F-stat	p-val
G	11	33853	1328	< 0.05
V	181	1386	54	< 0.05
R	7	11960	469	< 0.05
VG	1991	246	10	< 0.05
VR	1267	118	5	< 0.05
Residual	153790	25	1	NA
